# Mice Deficient of Glutamatergic Signaling from Intrinsically Photosensitive Retinal Ganglion Cells Exhibit Abnormal Circadian Photoentrainment

**DOI:** 10.1371/journal.pone.0111449

**Published:** 2014-10-30

**Authors:** Nicole Purrier, William C. Engeland, Paulo Kofuji

**Affiliations:** Department of Neuroscience, University of Minnesota, Minneapolis, Minnesota, United States of America; Morehouse School of Medicine, United States of America

## Abstract

Several aspects of behavior and physiology, such as sleep and wakefulness, blood pressure, body temperature, and hormone secretion exhibit daily oscillations known as circadian rhythms. These circadian rhythms are orchestrated by an intrinsic biological clock in the suprachiasmatic nuclei (SCN) of the hypothalamus which is adjusted to the daily environmental cycles of day and night by the process of photoentrainment. In mammals, the neuronal signal for photoentrainment arises from a small subset of intrinsically photosensitive retinal ganglion cells (ipRGCs) that send a direct projection to the SCN. ipRGCs also mediate other non-image-forming (NIF) visual responses such as negative masking of locomotor activity by light, and the pupillary light reflex (PLR) via co-release of neurotransmitters glutamate and pituitary adenylate cyclase-activating peptide (PACAP) from their synaptic terminals. The relative contribution of each neurotransmitter system for the circadian photoentrainment and other NIF visual responses is still unresolved. We investigated the role of glutamatergic neurotransmission for circadian photoentrainment and NIF behaviors by selective ablation of ipRGC glutamatergic synaptic transmission in mice. Mutant mice displayed delayed re-entrainment to a 6 h phase shift (advance or delay) in the light cycle and incomplete photoentrainment in a symmetrical skeleton photoperiod regimen (1 h light pulses between 11 h dark periods). Circadian rhythmicity in constant darkness also was reduced in some mutant mice. Other NIF responses such as the PLR and negative masking responses to light were also partially attenuated. Overall, these results suggest that glutamate from ipRGCs drives circadian photoentrainment and negative masking responses to light.

## Introduction

The SCN in the hypothalamus houses the central circadian pacemaker and regulates various circadian oscillators in peripheral tissues [1], [2], [3]. The interaction of the central SCN oscillator and peripheral circadian oscillators generates coherent, ∼24 hour, daily body rhythms of temperature, hormonal secretion, sleep-wake-cycles and cardiovascular function [1], [2]. Neurochemical signals from the retina provide all the photic information required for the precise alignment (entrainment) of the SCN pacemaker to environmental light-dark cycles. In mammals, this photic information is conveyed exclusively by a small subset of retinal ganglion cells that express the opsin-based photopigment melanopsin (Opn4) [4], [5], [6]. Additionally, melanopsin-expressing retinal ganglion cells (intrinsically photosensitive retinal ganglion cells or ipRGCs) also mediate other behaviors associated with organismal responses to ambient light levels [6], [7]. These NIF visual photoresponses include the PLR, negative masking responses to light, and acute suppression of melatonin secretion [4], [6], [7].

During the photoentrainment process of the SCN, light depolarizes ipRGCs via melanopsin-mediated phototransduction and outer retinal influences [5], [6]. ipRGCs then release the neurotransmitters glutamate and PACAP from their synaptic terminals to distinct brain regions [8]. Glutamatergic neurotransmission from retinal ganglion cells, including ipRGCs, is reliant on the storage of glutamate in synaptic vesicles by the vesicular glutamate transporter 2 (VGLUT2) [9], [10]. The highly homologous vesicular glutamate transporter 1 (VGLUT1) and vesicular glutamate transporter 3 (VGLUT3) are also expressed in the retina but in functionally distinct glutamatergic neurons [10], [11]. Indeed mice with genetic ablation of VGLUT1 have impaired neurochemical signaling from rods and cones to the inner retina but circadian photoentrainment and pupillary light reflexes are spared [10].

The presence of both glutamate and PACAP in ipRGC synaptic terminals raises the question on the relative role of each neurotransmitter system for various NIF visual behaviors. PACAP or PACAP receptor knockout mice show impaired photoentrainment [12], [13], and an altered pupillary light reflex [14], [15]. On the other hand, there is disagreement on whether PACAP signaling is required for negative masking responses to light [12], [14]. Moreover, PACAP signaling is required for light-induced stimulation of adrenal corticosterone secretion in mice [16]. More recently, selective ablation of VGLUT2 expression in ipRGCs in mice indicated that glutamate is required for aversion of light at neonatal stages but not for the PLR [17]. Here we investigate the role ipRGC glutamatergic neurotransmission for circadian photoentrainment and other NIF photoresponses in mice. We found that ablation of VGLUT2 expression in ipRGCs profoundly impairs, but does not abolish circadian photoentrainment, negative masking responses to light, and the PLR. Moreover, some mutant mice had diminished circadian locomotor activity, with bouts of activity occurring across all circadian times. These findings suggest a complementary and non-redundant role of PACAP and glutamate in ipRGCs for various NIF visual behaviors.

## Materials and Methods

### Animals

Conditional ablation of VGLUT2 in mice was performed in our laboratory by crossing *Vglut2^flx/flx^* mice in which two loxP elements were introduced into the mouse genome flanking exon 2 of the *Vglut2* (*Slc17a6*) locus [18] with *Opn4^Cre/Cre^* mice in which Cre recombinase is expressed selectively in ipRGCs [19](kindly provided by Dr. Samer Hattar). Mutant mice *Opn4^Cre/+^, Vglut2^flx/flx^* were used in all experiments while littermates in which Cre recombinase was absent served as controls. Mice had access to food and water *ad libitum*. This study was carried out in strict accordance with the recommendations in the Guide for the Care and Use of Laboratory Animals of the National Institutes of Health. The experiments were approved by the Institutional Animal Care and Use Committee at the University of Minnesota, and adhered to the Association for Research in Vision and Ophthalmology (ARVO) guidelines for animal use in vision research. Prior to tissue collection, mice were humanely euthanized by isoflurane anesthesia followed by cervical dislocation and all efforts were made to minimize suffering. Both male and female adult (postnatal days 21 to 90) mice were used in all experiments.

### Immunocytochemistry

Immunocytochemistry was performed essentially as previously described [20], [21]. Mice were anesthetized with isoflurane and perfused transcardially with 1× phosphate-buffered saline (PBS), pH 7.4, followed by 20 ml of 4% paraformaldehyde in PBS. The brains and eyes were extracted, fixed overnight in the same fixative at 4°C, and cryoprotected in 30% (w/v) sucrose in PBS at 4°C until they sank to the bottom. Each brain was sectioned at 100 µm on a Vibratome while retinal tissue was sectioned at 15 µm in a cryostat. Sections were incubated in PBS containing 0.5% Triton X-100 and 10% goat serum overnight to block nonspecific labeling. Retinal sections were then incubated with primary antibodies at 4°C overnight, washed and incubated overnight with secondary antibodies and DAPI (Roche Diagnostics, Indianapolis, IN) to label nuclei. For brain sections, the incubation periods were extended to two-three days for primary antibodies and one day for secondary antibodies. The primary antibodies used in this study were: rabbit anti-melanopsin (1∶500)[21], guinea pig anti- VGLUT2 (Synaptic Systems, Gottingen, Germany,1∶1000), guinea pig anti-VGLUT1 (Synaptic Systems, 1∶1000), guinea pig anti- VGLUT3 (Synaptic Systems, 1∶1000), rabbit anti-VIP (Vasoactive Intestinal Peptide) (Immunostar, Hudson, WI, 1∶1000). We used goat anti-rabbit Alexa-488 and goat anti-guinea pig Alexa-594 as secondary antibodies (Invitrogen, Grand Island, NY, 1∶750). Retinas and brain slices were stained in parallel to ensure specificity of antibody labeling. Confocal z stacks were acquired using an Olympus FluoView FV1000 confocal microscope with 20X, 40X, or 60X oil-immersion objectives. Editing of images was limited to adjusting the brightness and contrast levels using ImageJ software (NIH, Bethesda). Experiments were replicated with a minimum of 3 individual mice. Localization and morphology of the stained cells with each primary antibody matched previous descriptions in retina and SCN [10], [11], [21], [22], confirming the specificity of the antibodies used in this study.

### Circadian Locomotor Activity

Mice (6–12 weeks of age) were housed within a temperature-controlled facility in individual cages equipped with 4.5-inch running wheels that were maintained in ventilated chambers that permitted control of the light-dark cycle. The animals were maintained on a 12-h light (∼900 lux, white fluorescent tube light): 12-h dark (LD 12:12) cycle for at least 3 weeks and then transferred to the ventilated chambers. Manipulation in DD conditions were performed under dim red light. Abrupt 6-h advances in the LD schedule were achieved by advancing the time of lights-on (light phase) and shortening the time of lights-off (dark phase). Conversely, abrupt 6-h delays in the LD schedule were achieved by delaying the time of lights-on, and lengthening the time of lights-off. After the 6 h advance or delay of the LD cycle, the number of days to re-entrain was defined for each animal as the number of days required to shift activity by 6±0.5 h. Negative masking responses to light was assessed by recording running wheel activity for mice submitted to a T7 (3.5-h light: 3.5-h dark) cycle [23] over several days. Activity data were recorded continuously by a PC system and displayed and analyzed by using commercial software (Chronobiology Kit, Stanford Software Systems). The free-running period was calculated (days 1–10 in DD) by using a χ2 periodogram [24]. The amplitude of the circadian component was estimated from a normalized Fourier spectrum by using 10 days in DD. Original data were collected at one-min intervals. We grouped the data for male and female mice as we did not observe statistically significant differences between sexes for locomotor activity circadian period or circadian amplitude.

### Intravitreal cholera toxin injections

Mice were anesthetized by intraperitoneal injection of a solution containing ketamine (100 mg/ml) and xylazine (10 mg/ml) at a dose of 0.1 ml/100 g body weight. The vitreous body of the left eye was injected with 2 µl of a 0.1% solution of cholera toxin B protein (CTB) conjugated to Alexa Fluor-488 (Invitrogen) in sterile PBS. Following the injection, the needle was held in place for 30 sec to prevent leakage. Mice were perfused intracardially 2 days after the injection.

### Pupillary Light Response Measurements

After overnight dark adaptation, mice were restrained by scruff immobilization [25] and one eye was monitored under infrared light with a Sony DCR-HC96 camera (Sony, Japan) fitted with an infrared filter. PLRs were evoked by 20 seconds of a low (4 µW/cm^2^) or high intensity white light (3.8 mW/cm^2^) stimulus. Light stimuli were produced by a 300-W Xenon Arc lamp light source (Sutter Instruments) and delivered through a liquid light guide. A filter wheel with neutral density filters and a Lambda 10–3 optical filter changer with SmartShutter (Sutter Instruments, Novato, CA) was used to control the intensity and duration of light. Video frames were captured for 20 seconds prior to the application of light and during the 20-second light stimulus. Pupillary area was measured from video images prior to the onset of light and at the end of the 20-second light stimulus using Image J software. Pupillary areas after illumination were expressed relative to the area of the pupil in darkness. All experiments were conducted during the light period of 12∶12-hour light-dark cycles.

### Statistical Analyses

Statistical analyses were performed using OriginPro 8.1 (MicroCal). Statistical comparison of means was performed using a Student's t test. Comparisons of variance were performed using two-sample test for variance. Significance was concluded when p<0.05. Data are presented as mean ± SE.

## Results

### Lack of VGLUT2 expression in Vglut2-cKO ipRGCs

**Figure 1:**
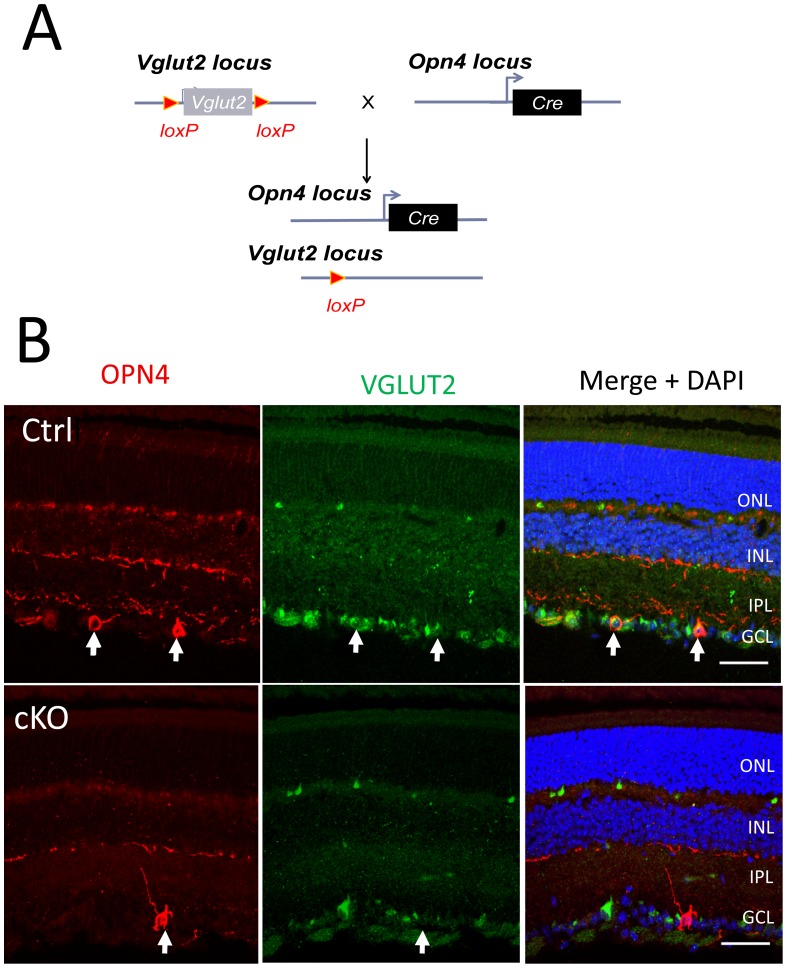
Ablation of VGLUT2 expression in Vglut2-cKO mouse retinas. (A) Diagram of gene targeting strategy. Mouse with exon 2 of *Vglut2* gene flanked by LoxP sites was bred with the Opn4-Cre mouse line in which Cre was inserted in the Opn4 gene locus. Progeny contained mice with deletion of Vglut2 exon 2 in ipRGCs (Vglut2-cKO mice). (B) Retinal sections of Vglut2-cKO and control littermate stained for Opn4, VGLUT2. Notice the co-localization of VGLUT2 with Opn 4 (arrows) in the control but not in the Vglut2-cKO mice retinas. ONL, outer nuclear layer; INL, inner nuclear layer; IPL, inner plexiform layer; GCL, ganglion cell layer. Scale bar  = 50 µm.

To examine the role of ipRGC glutamatergic neurotransmission for circadian photoentrainment and other NIF visual functions, we performed the conditional ablation of VGLUT2 in ipRGCs. Previous studies specified that VGLUT2 is the only vesicular transporter required for glutamate storage in synaptic vesicles of RGCs including ipRGCs [10], [17]. Ablation of VGLUT2 expression in ipRGCs was achieved by breeding *Opn4^Cre/Cre^* mice with *Vglut2^flx/flx^* mice (Figure 1A). The *Opn4^Cre/+^, Vglut2^flx/flx^* mice (hereafter designated as Vglut2-cKO) developed with no apparent gross behavioral abnormalities. Retinal projections to the brain in the Vglut2-cKO mouse line were apparently normal as assessed by unilateral intravitreal injection of cholera toxin B protein (CTB). CTB-labeled terminals were detected bilaterally in the SCN (Figure S1). In the lateral geniculate nucleus (LGN) of the thalamus, the dorsal domain is innervated mostly by retinal afferents from conventional RGCs while the ventral domain and the intergeniculate leaflet (IGL) receive afferents mostly from ipRGCs [26]. CTB labeling in the ventral domain of the LGN and the IGL indicated that ipRGC innervation is preserved in the Vglut2-cKO mouse (Figure S1). Other NIF visual centers such as the olivary pretectal nucleus, superior colliculus, peri-supraoptic nucleus, and subparaventricular zone of the hypothalamus were also labeled with CTB (data not shown). We also did not detect major structural changes in the SCN as assessed by vasoactive intestinal peptide (VIP) (Figure S2). In summary, ipRGCs in the Vglut2-cKO mouse innervate the expected target brain centers, and the SCN does not show obvious structural abnormalities.

VGLUT2 expression in the rodent retina is mostly confined to RGCs and in a subset of cone pedicles [10], [11], [27]. Although VGLUT2 expression is concentrated in neuronal synaptic terminals, cell body expression can also be detected by immunocytochemistry [11]. Immunostaining of Vglut2-cKO and control littermate retinas for VGLUT2 shows most of the staining confined to the ganglion cell layer and distinct puncta in the outer plexiform layer (Figure 1B). The overall morphology of the retinal layers was similar in control and mutant mice. In control mouse retinas, co-labeling of VGLUT2 and melanopsin shows the expression of VGLUT2 in ipRGCs. However, VGLUT2 expression is ablated in ipRGCs from Vglut2-cKO retinas (Figure 1B). Co-labeling of Vglut2-cKO retinas for VGLUT1 or VGLUT3 and melanopsin does not show their expression in ipRGCs (Figures S3 and S4). Our analysis of VGLUT expression is consistent with the lack of expression of VGLUT2 expression in ipRGCs from Vglut2-cKO mice retinas and lack of up regulation of VGLUT1 and VGLUT3 in these cells.

### Circadian photoentrainment is disrupted in the Vglut2-cKO mouse

**Figure 2:**
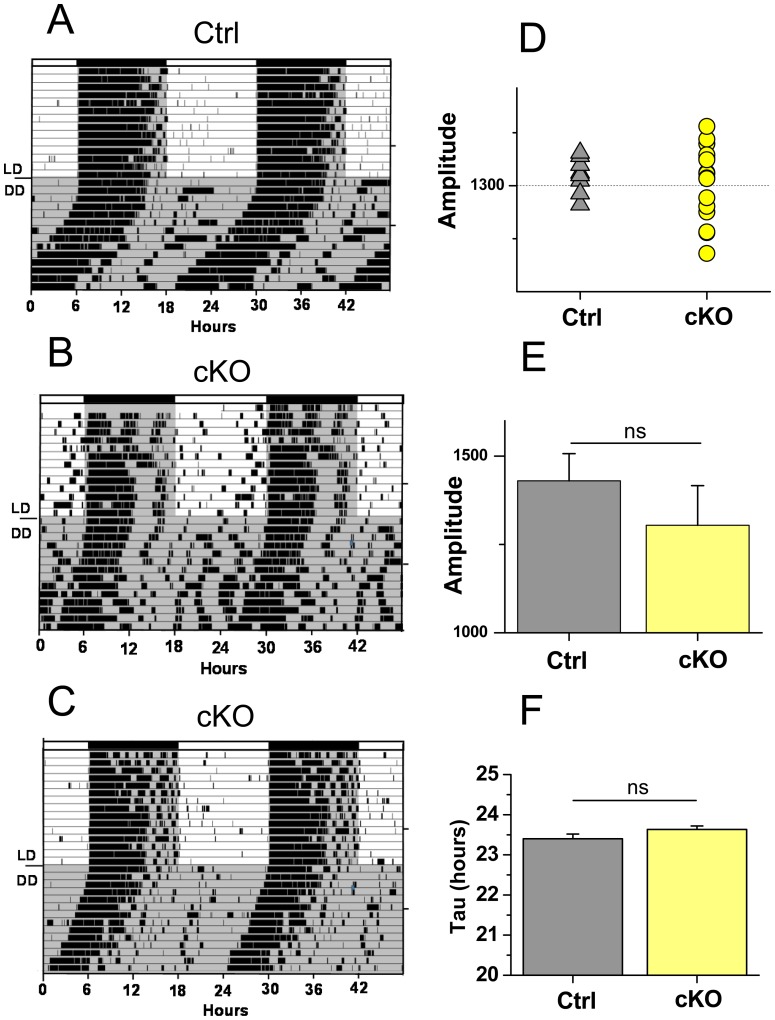
Circadian locomotor activity for the Vglut2-cKO and control mice under LD and DD conditions. (A–C) Representative activity records from animals initially held in a 12:12 LD cycle then transferred to DD. Control (Ctrl)(A) and mutant (Vglut2-cKO)(B–C) animals demonstrate entrainment in LD as indicated by enhanced activity in the dark period. In DD the animals show a free running activity rhythm with some Vglut2-cKO mice exhibiting low-amplitude rhythms (B). In these animals, although the majority of activity was confined to the dark phase under LD, activity onset in DD was variable. Shaded regions indicate periods of darkness mirrored in LD bars above the actograms. (D–E) Amplitude of locomotor activity in DD was more variable among Vglut2-cKO (n = 17) than control (n = 9) mice. (F) Period of locomotor activity was comparable between control (n = 9) and Vglut2-cKO (n = 17) mice.

We first analyzed the characteristics of the circadian activity pattern of Vglut2-cKO mice and their respective control littermates. The general pattern of activity in a light regimen of 12 h lights on and 12 h lights off (LD) condition was similar in both genotypes, with bouts of activity occurring after lights off, which lasted for several hours (Figures 2 A–C). However, some Vglut2-cKO mice showed a more irregular pattern of circadian locomotor activity with some running even with lights on (Figure 2B). To reveal the endogenous circadian locomotor activity, we transferred the mice to constant dark conditions (DD). The general pattern of activity in DD condition was also equivalent in both mice genotypes with most mice demonstrating a free-running rhythm. However, as under LD conditions, some mutant mice showed irregular bouts of running activity during all phases of circadian time (Figure 2B). Control mice used in this experiment had an average free-running period of 23.40±0.12 h (n = 9), whereas the Vglut2-cKO group averaged 23.63±0.09 h (n = 17). Collectively, the Vglut2-cKO mice group displayed a trend for more variable circadian rhythmicity than the control group in DD conditions although differences in amplitude variance of the wheel-running rhythm did not reach statistical significance (Figures 2D–E).

**Figure 3:**
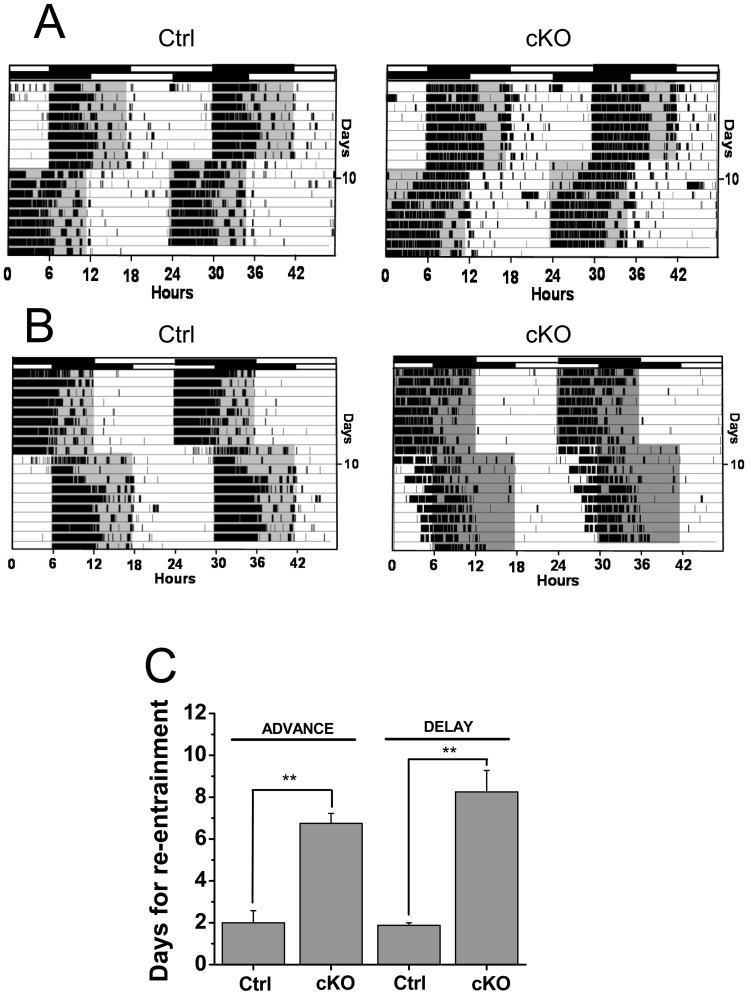
Vglut2-cKO mice show impaired re-entrainment to phase shifts in the LD cycle. (A) Representative double-plotted actograms of mice subjected to 6 hr phase advance on days marked. Top bars indicate initial LD cycle; bottom bars below indicate shifted cycle. (B) Representative double-plotted actograms of mice subjected to 6 hr phase delay on days marked. Top bars indicate initial LD cycle; bottom bars below indicate shifted cycle. (C) Number of days required for reentrainment after the 6 hr phase advance or phase delay in control (n = 7) and Vglut2-cKO (n = 4) mice. Vglut2 mice showed delayed re-entrainment to either phase advances or phase delays in the LD cycles (** p<0.05)).

The impairment in photoentrainment of the Vglut2-cKO mice also was reflected by difficulties in readjusting to a ‘jet lag’ light-dark cycle produced by 6 h advanced and delayed light onsets (Figure 3). In the control mice, this phase advance evoked a rapid shift of locomotor activity rhythms, which took 2.0±0.6 days (n = 7) to re-entrain to the new LD regimen (Figures. 3A and 3C). In contrast, in the Vglut2-cKO mice, the phase advance in light onset required 6.8±0.5 days (n = 4) for reentrainment. Similarly, slower reentrainment was observed for the Vglut2-cKO mice in response to a 6 h phase delay (Figures 3B and 3C).

**Figure 4:**
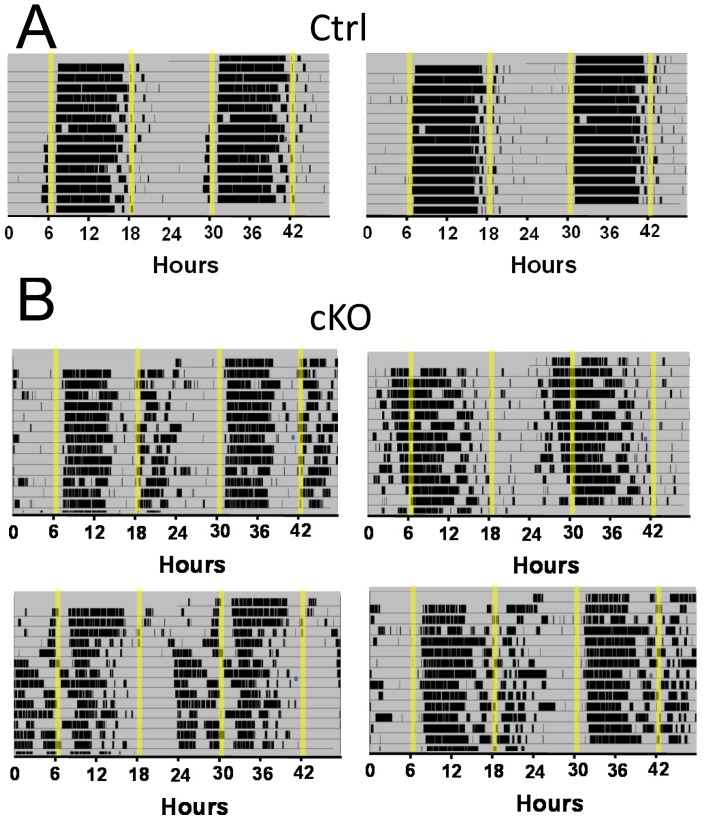
Vglut2-cKO mice exhibit abnormal entrainment to a skeleton photoperiod. Representative actograms from control (A) and Vglut2-cKO animals (B) under skeleton photoperiod light cycles (1 hour light pulses, 12 h apart). Control mice photoentrain to the 1-h light pulses and restrict their activity to one of the dark periods. Vglut2-cKO mice showed a variable degree of photoentrainment with some activity in both dark periods. Shaded regions of the activity records indicate periods of darkness.

The variable circadian rhythmicity in DD for the Vglut2-cKO mice precluded us from performing light pulse experiments at defined circadian times to establish a phase response curve. Instead, to further evaluate circadian photoentrainment, we subjected the mice to a skeleton photoperiod in which light pulses of one hour duration were given 11 h apart to simulate dawn and dusk light exposure in nocturnal animals [28]. Under these conditions, it was expected that the locomotor activity would consolidate in one of the two dark periods between light pulses with inactivity in the opposite dark period. Indeed the control mice entrained to these light pulses with locomotor activity largely restricted to one of the dark periods (Figure 4A). The Vglut2-cKO mice, on the other hand, displayed variable photoentrainment with some mice showing extensive locomotor activity across both dark periods (Figure 4B).

### PLR and negative masking responses to light are impaired in the Vglut2-cKO mouse

**Figure 5:**
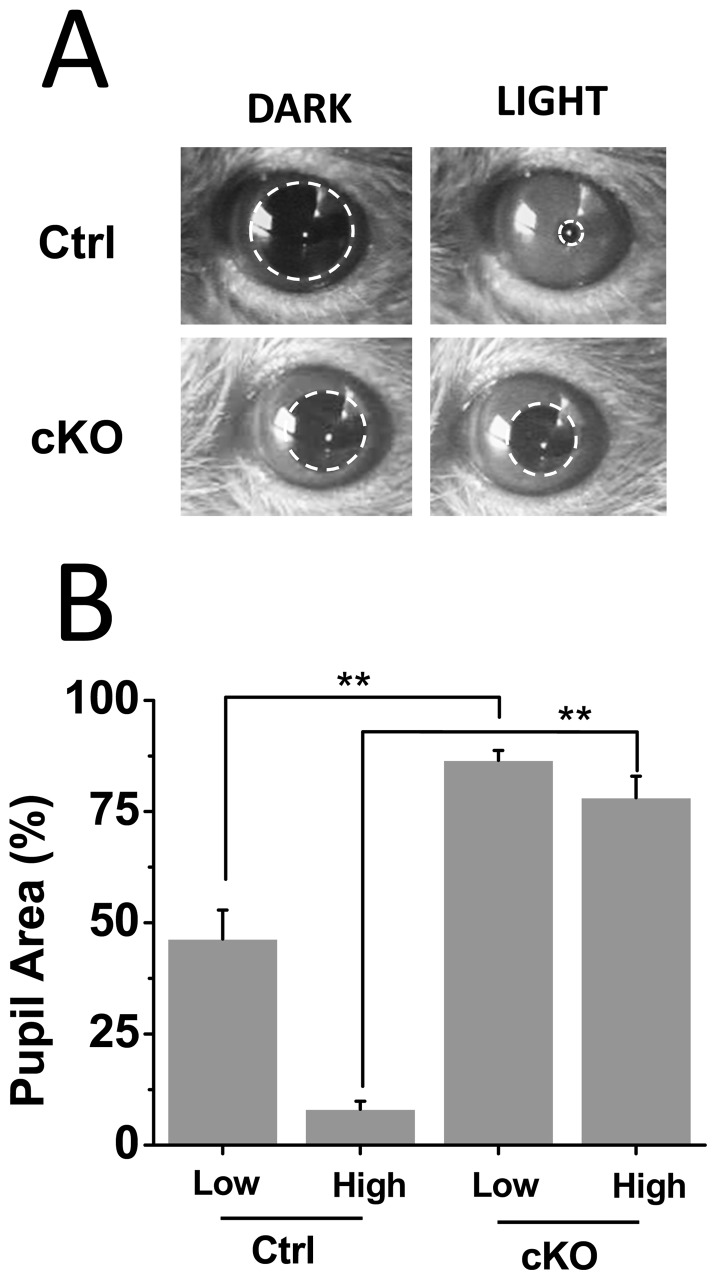
Attenuated PLRs in Vglut2-cKO mice. (A) Images of control and Vglut2-CKO mice pupils before and during exposure to high intensity (3.8 mW/cm^2)^ white light stimuli. (B) Summary of PLRs measured in control mice. Consensual PLRs were measured in control (n = 8) and Vglut2-cKO (n = 10) mice. Vglut2-cKO mice show severe deficits in PLRs under low (4 µW/cm^2)^ and high (3.8 mW/cm^2)^ intensity white light stimuli (** p<0.05). Light stimuli were delivered for 20 s and maximum pupil area was measured before and during the light stimulus. Percent of pupil area following the light stimulus is shown normalized to the pupil area during dark conditions.

Lack of fast glutamatergic signaling from ipRGCs would be expected to impair NIF visual functions attributed to these cells such as the pupillary light reflex (PLR) and negative masking responses to light [29], [30]. We first measured the consensual PLR in these mice at two light intensities (high light intensity  = 3.8 mW/cm^2^ and low light intensity  = 4 µW/cm^2^). PLR was evoked by a white light stimulus of 20 seconds duration. In control littermate mice, the pupillary area constricted to ∼8% under high light intensity (n = 8) and ∼46% under low light intensity (n = 8) in comparison to prestimulus conditions (Figure 5A–B). In contrast, the Vglut2-cKO mice showed a much attenuated PLR. The pupillary area constricted to only 78% under high light intensity (n = 10) and 86% under low light intensity (n = 10). Application of 100 mM carbachol in the mutant mouse eye (n = 3) was able to elicit full pupillary constriction (data not shown), indicating that lack of light-evoked responses was not due to malformation of the pupillary constriction apparatus.

**Figure 6:**
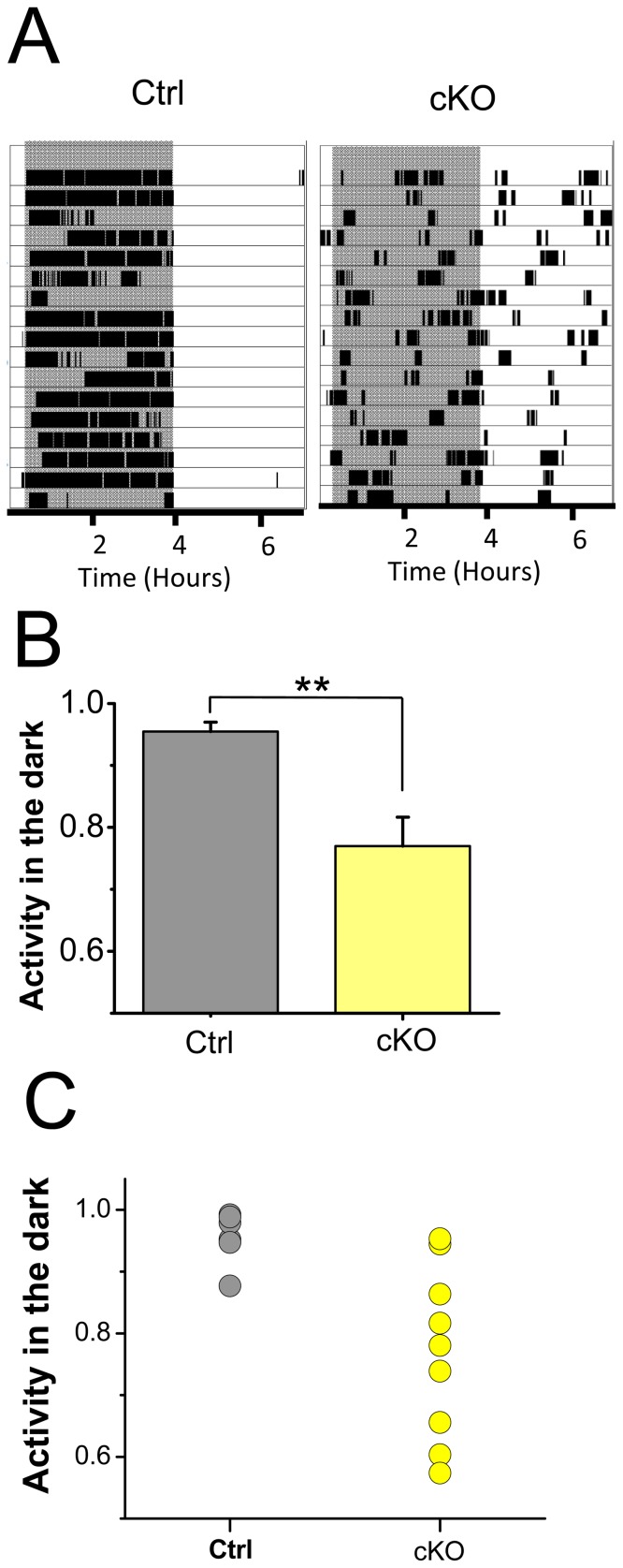
Negative masking responses to light are impaired in the Vglut2-cKO mice. Control and Vglut2-cKO mice were subjected to 3.5 h light: 3.5 h dark cycles for 14 days. (A) Control mice show robust activity during dark period reflecting strong aversion to locomotor activity under bright light conditions. The Vglut2-cKO mice, on the other hand, exhibited activity in both dark and light periods indicative of less pronounced negative masking responses to light. Shaded regions indicate periods of darkness. (B) Activity in the dark normalized to the total activity is higher in the control (n = 7) than the Vglut2-cKO (n = 8) mice (** p<0.05). (C) Analysis of the individual mice shows more variable masking responses among the Vglut2-cKO mice (n = 9) than control littermates (n = 7).

In line with the PLR results, we also observed major deficiencies in negative masking responses to light. Bright light suppresses locomotor activity in nocturnal animals including mice [31]. Running wheel activity was recorded for two weeks in mice kept on 3.5:3.5 light/dark (LD) cycles to measure effects of light independent of circadian rhythms [23]. This light cycle assesses negative masking, because it is difficult for the mice to entrain to light cycles that move across the circadian cycle [23]. By plotting running activity for a period of 7 h for the control and Vglut2-CKO mice, it is apparent that their locomotor activity in the dark and light periods differ (Figure 6A). While the control mice restricted their activity to the dark periods of the ultradian cycle (activity in the dark period/total activity  = 0.95±0.01, n = 7), the Vglut2-cKO mice show activities across the light-dark cycles (activity in the dark period/total activity  = 0.77±0.04, n = 9) demonstrating significant impaired negative masking responses to light (Figure 6B). Heterogeneity of responses to light was prominent among the Vglut2-cKO mice (Figure 6C). While some mutant mice showed almost no preference for locomotor activity in the dark, other mice showed locomotor activity restricted to the dark periods (Figure 6C).

## Discussion

Our study suggests the fundamental role of glutamatergic neurotransmission from ipRGCs to elicit light-driven NIF behaviors in mice. The results show that selective silencing or knock-down of glutamatergic signaling from ipRGCs leads to markedly diminished pupillary responses to light, impaired negative masking responses to light, and variable but diminished light entrainment of circadian locomotor activity. In constant darkness, a number of Vglut2-cKO animals also showed diminished locomotor rhythmicity with bouts of activity across all circadian times. Despite functional deficits, the retina and the SCN did not show obvious structural abnormalities.

The phenotype observed in this study is in sharp contrast to those observed upon genetic ablation of ipRGCs in which various NIF behaviors evoked by light were almost completely suppressed [29], [30]. In terms of circadian locomotor activity, ablation of ipRGCs in adult mice leads to a “free running” phenotype in which environmental light has little effect on the circadian clock [29], [30]. Circadian rhythmicity is however maintained in these animals, indicating a fully functional endogenous circadian pacemaker. Similar findings are also reported for mice in which phototransduction is absent in all photoreceptors in the retina [32]. In contrast, we found that blocking glutamatergic neurotransmission only partially decouples the endogenous pacemaker from the retinal signals. While the Vglut2-cKO mice displayed longer latencies to adjust to changes in environmental light as evidenced by the “jet lag” experiments, the mice eventually entrained to the novel light regimen. Likewise, the Vglut2-cKO mice showed partial entrainment to the imposed skeleton photoperiod, indicating partial signaling of ipRGCs to various circadian brain centers. These results seem to indicate a residual signaling of ipRGCs in the Vglut2-cKO mice, most likely, via release of the peptide PACAP. There is increasing evidence that glutamate and PACAP have a synergistic role in the light entrainment pathway from the retina to the SCN. Glutamatergic agents have an effect *in vitro* and *in vivo* that closely mimics the effect of light in the rat suprachiasmatic nucleus [33], [34]. PACAP exerts effects on the SCN *in vitro* and *in vivo* that are also consistent with function as an entrainer signal [35], [36] or modulator of the glutamate-mediated light-entrainment [37]. Moreover, PACAP-deficient mice exhibit significant impairment of light-induced phase shifts in circadian locomotor activity [12], [14] in agreement with the notion that both glutamatergic and PACAP-mediated neurotransmission from ipRGCs are required for effective synchronization of the SCN to environmental light-dark cycles.

The relative role of glutamate and PACAP for signaling from ipRGCs beyond photoentrainment is much less clear. Negative masking responses to light is one behavioral output that is driven by ipRGCs light signals, but there is controversy on whether PACAP-deficient mice have impaired negative masking responses to light [12], [14]. Our results clearly demonstrate that lack of glutamatergic transmission diminishes the negative masking by light. A similar scenario was observed for another NIF visual behavior investigated, pupillary constriction in response to light. Light exposure even at high intensities was unable to elicit the degree of pupillary constriction in Vglut2-cKO mice seen in the littermate controls. This finding is in agreement with a recent study that also made use of conditional ablation of VGLUT2 expression in ipRGCs [17].

The present study has two potential limitations. First, it is not clear that suppression VGLUT2 extends to all ipRGCs in the VGlut2-cKO mice. The Opn4-Cre mouse line used in this study has been used previously for expression of reporter proteins in ipRGCs [19]. Cre-mediated expression of reporter proteins in all ipRGC subtypes [19] suggests that VGLUT2 expression should be downregulated or ablated across ipRGC subtypes. However we cannot discard the possibility that residual expression of VGLUT2 may account for some of the observed photic responses in the mutant mice. We think this is unlikely as another study using equivalent mice [17] has shown the complete suppression of ipRGC-dependent NIF behaviors such as negative phototaxis and light-induced distress vocalizations in neonatal mice [17]. Moreover, genetic ablation of ipRGCs using a similar Opn4-Cre mouse line shows the complete lack of NIF responses such as circadian photoentrainment and PLRs [29] pointing to effective expression of Cre recombinase in all melanopsin-expressing retinal ganglion cells. The second potential limitation is the potential ectopic expression of Cre recombinase in a small subset of brain neurons as described by Ecker et. al. [19]. The presumed ectopic expression of Cre recombinase in the Opn4-Cre mouse line seems restricted to non-visual areas such as some neocortical pyramidal cells and some neurons in the piriform cortex [19]. Nonetheless it seems improbable that loss of glutamatergic transmission in a subset of brain neurons unrelated to visual processing would account for the residual light photosensitivity the mutant Vglut2-cKO mice. We believe that the most parsimonious explanation for our results is that glutamatergic transmission is essential but not sufficient for the the NIF visual behaviors such as circadian photoentrainment.

Although our findings seem to indicate that NIF visual behaviors are dependent on the combined effect of glutamate and PACAP from ipRGCs on various brain targets, this is not necessary the rule as neonatal photoaversion (light-induced phototaxis and light-induced distress vocalizations) appears to be dependent only on glutamatergic neurotransmission from ipRGCs [17]. Conversely, in the PACAP knockout mouse there is a lack of light-induced elevation of renal sympathetic nerve activity and adrenal corticosterone secretion [16]. Thus an important quest is to delineate the relative role of each neurotransmitter system for the ever increasing types of behaviors or physiological outputs that ambient light influences [17], [19], [29].

An interesting and unexpected finding in our study was that a limited set ofVGlut2-cKO mice exhibited low-amplitude locomotor rhythms in constant darkness (DD) with marked activity during subjective day. This observation might indicate that glutamatergic signaling from ipRCGs is important for shaping normal development of circadian function. Previous work that addresses this possibility has been inconclusive. Bilateral eye enucleation in hamsters at early stages of postnatal development leads to animals with “free running” behavior in which the circadian rhythmicity is largely intact, suggesting that photic signals from the retina play a minor role in the postnatal development of the SCN [38]. Likewise, mice in which the majority of the retino-hypothalamic tract (RHT) is absent due to the lack of expression of the transcription factor Math 5 also “free run” and don't respond to photoentrainment signals [39]. Finally, ablation of ipRGCs by their selective expression of diphtheria toxin (DTA) also yields animals (Opn4-DTA) with endogenous circadian periodicity [30]. Collectively, these results suggest a minor or no role of the RHT in the development of the SCN. However, most of these studies were performed at postnatal stages so a role of RHT signal at prenatal stages cannot be excluded. Furthermore, partial signaling from the retina to the SCN in the Math 5 or Opn4-DTA mice is likely as 3–17% of ipRGCs remain in these mouse lines [30], [40]. The counter argument that RHT exerts a role in the establishment of circadian rhythmicity is derived from studies performed in the anophthalmic mice in which the eyes do not develop. In the anophthalmic mice there are conspicuous effects on the circadian rhythmicity with subpopulations of mice with unstable circadian periods and arrythmicity [41].

The reduced circadian rhythmicity in locomotor activity in a subset of cKO mice suggest that ipRGC glutamatergic signaling may influence the proper development of circadian rhythmicity per se. Lack of retinal signaling to the SCN during an early critical period could result in substantial and permanent changes in neuronal circuitries that underlie circadian rhythmicity. Such influence of the retina to the development of the SCN pacemaker function is supported by the finding that exposing perinatal mice to different seasonal photoperiods (either short or long days days) persistently alters the SCN neuronal rhythms [42].

In summary, we show that genetic knockdown or knockout of fast glutamatergic transmission from ganglion cell photoreceptors results in partial suppression of NIF visual responses such as circadian photoentrainment and negative masking responses to light. Efferent projections from ganglion cell photoreceptors were preserved in the mutant mice that showed no clear structural abnormalities. The residual light-evoked responses suggest the complementary role of the co-neurotransmitter PACAP for these behaviors.

## Supporting Information

Figure S1 **Deletion of VGLUT2 expression does not affect retinal projections to the SCN and LGN.** (A) Control, (B) Vglut2-cKO SCNs were labeled by intraocular injection of fluorescently conjugated CTB. (C) Control, (D) Vglut2-CKO contralateral LGNs were labeled upon intraocular injection of fluorescently conjugated CTB. dLGN are labeled ‘d’, vLGN are labeled ‘v’ and arrows indicate IGL. Notice the projections to dLGN, vLGN and IGL. Scale bar  = 100 µm for (A to D). CTB, cholera toxin B subunit; dLGN, dorsal LGN; IGL, intergeniculate leaflet; LGN, lateral geniculate nucleus; vLGN, ventral LGN.
(TIF)

Figure S2 **Immunostaining for VIP suggests structural integrity of Vglut2-cKO SCN.** (A) Control, (B) Vglut2-cKO SCNs were labeled with VIP. Optic chiasm is labeled ‘oc’, third ventricles are labeled ‘3v’. Scale bar  = 100 µm.
(TIF)

Figure S3 **Expression of VGLUT1 is not altered in Vglut2-cKO retinas.** Immunostaining for Opn4 and VGLUT1 in (A) control and (B) Vglut2-cKO retinas. Most of VGLUT1 expression is found in the IPL and ONL. VGLUT1 is not found in ipRGCs from either mutant or control retinas (arrows). ONL, outer nuclear layer; INL, inner nuclear layer; IPL, inner plexiform layer; GCL, ganglion cell layer. Scale bar  = 50 µm.
(TIF)

Figure S4 **Expression of VGLUT3 is not altered in Vglut2-cKO retinas.** Immunostaining for Opn4 and VGLUT3 in (A) control and (B) Vglut2-cKO retinas. Most of VGLUT3 expression is found in the IPL. VGLUT3 is not found in ipRGCs from either mutant or control retinas (arrows). ONL, outer nuclear layer; INL, inner nuclear layer; IPL, inner plexiform layer; GCL, ganglion cell layer. Scale bar  = 50 µm.
(TIF)
