# Supplementary figures and images for: Mice Deficient of Glutamatergic Signaling from Intrinsically Photosensitive Retinal Ganglion Cells Exhibit Abnormal Circadian Photoentrainment
Source: PLoS One. 2014 Oct 30;9(10):e111449. doi: 10.1371/journal.pone.0111449 (PMC4214747; doi:10.1371/journal.pone.0111449)

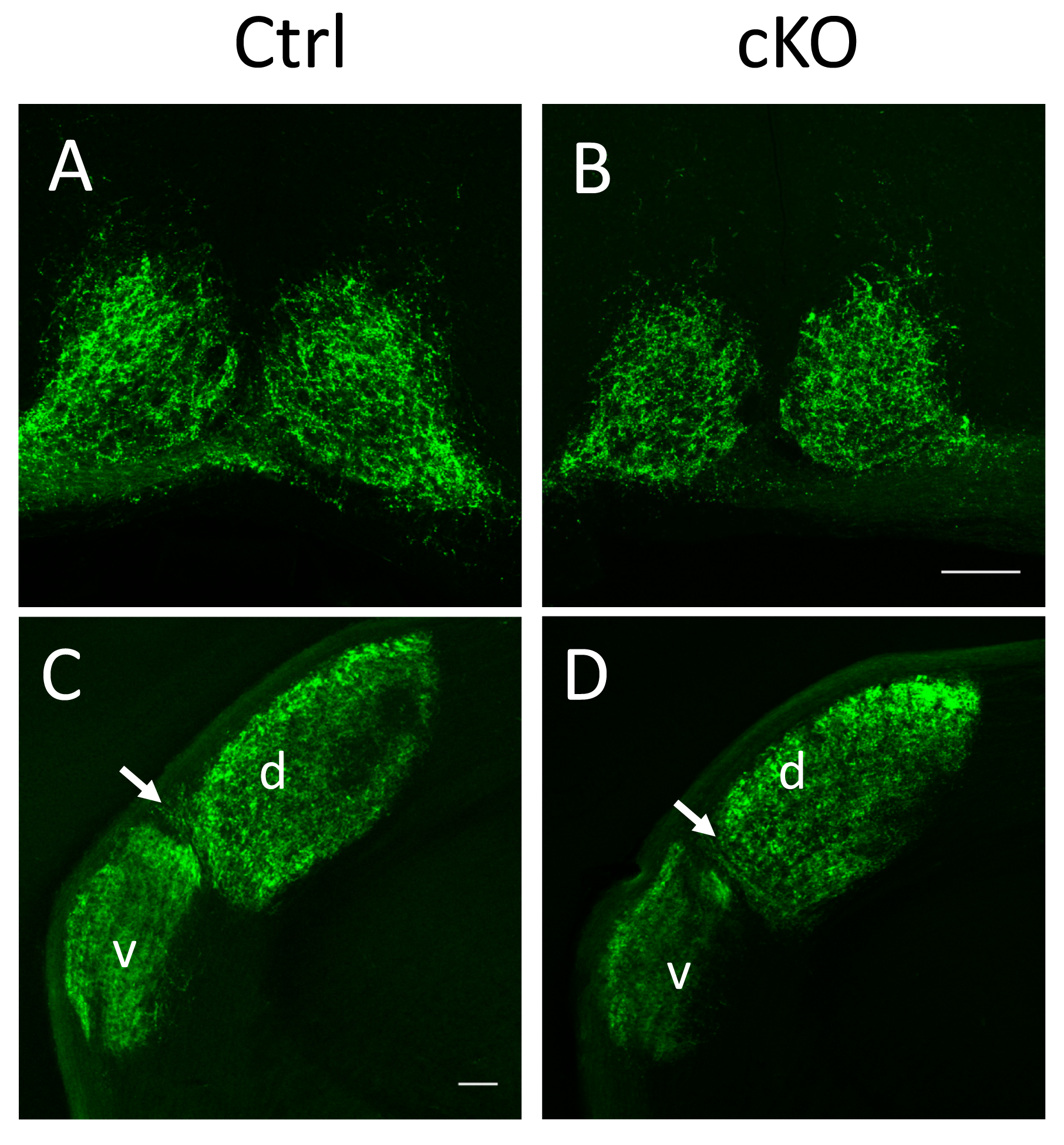

Supplement: Figure S1 — Deletion of VGLUT2 expression does not affect retinal projections to the SCN and LGN. (A) Control, (B) Vglut2-cKO SCNs were labeled by intraocular injection of fluorescently conjugated CTB. (C) Control, (D) Vglut2-CKO contralateral LGNs were labeled upon intraocular injection of fluorescently conjugated CTB. dLGN are labeled ‘d’, vLGN are labeled ‘v’ and arrows indicate IGL. Notice the projections to dLGN, vLGN and IGL. Scale bar = 100 µm for (A to D). CTB, cholera toxin B subunit; dLGN, dorsal LGN; IGL, intergeniculate leaflet; LGN, lateral geniculate nucleus; vLGN, ventral LGN. (TIF) [file pone.0111449.s001.tif]

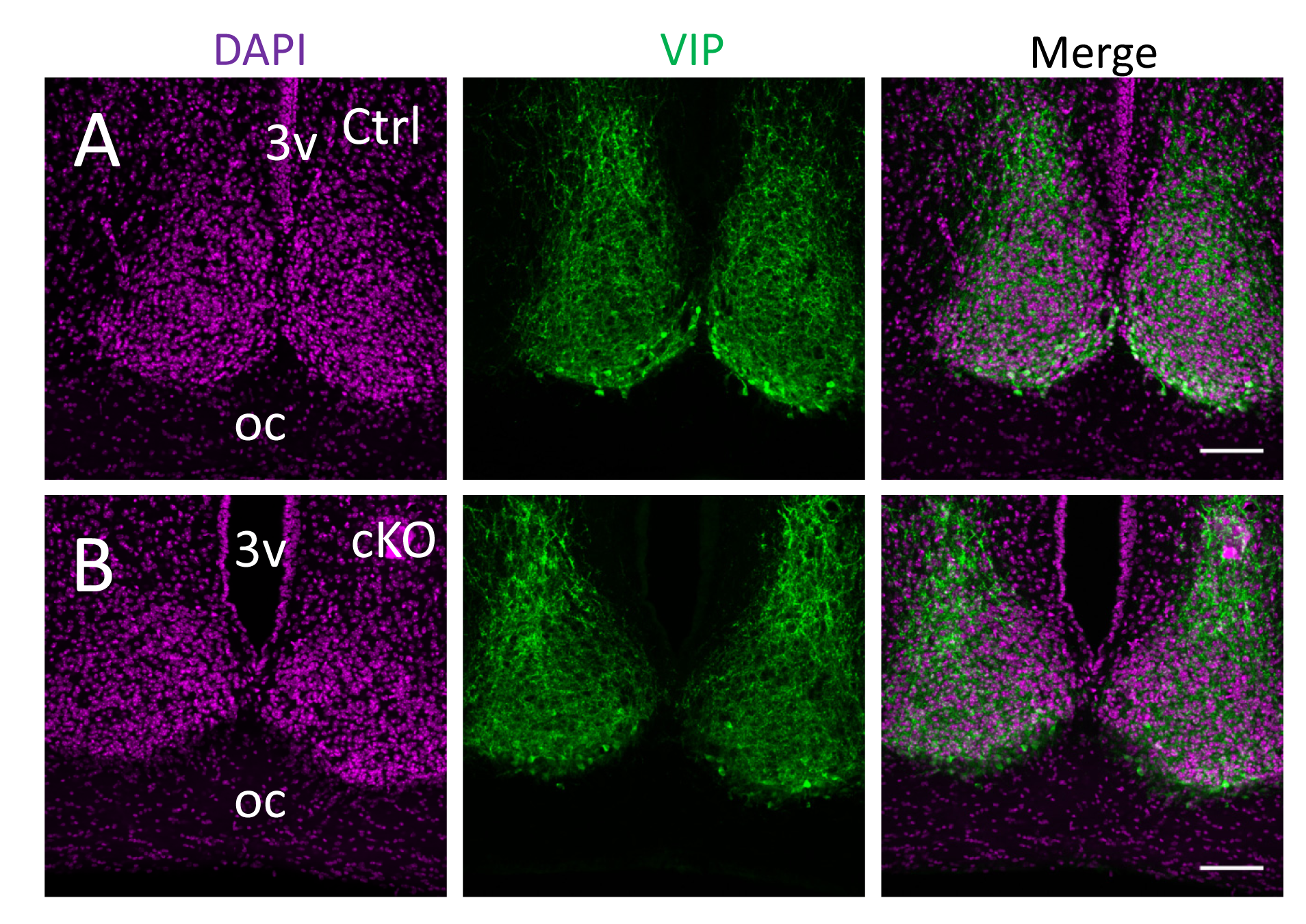

Supplement: Figure S2 — Immunostaining for VIP suggests structural integrity of Vglut2-cKO SCN. (A) Control, (B) Vglut2-cKO SCNs were labeled with VIP. Optic chiasm is labeled ‘oc’, third ventricles are labeled ‘3v’. Scale bar = 100 µm. (TIF) [file pone.0111449.s002.tif]

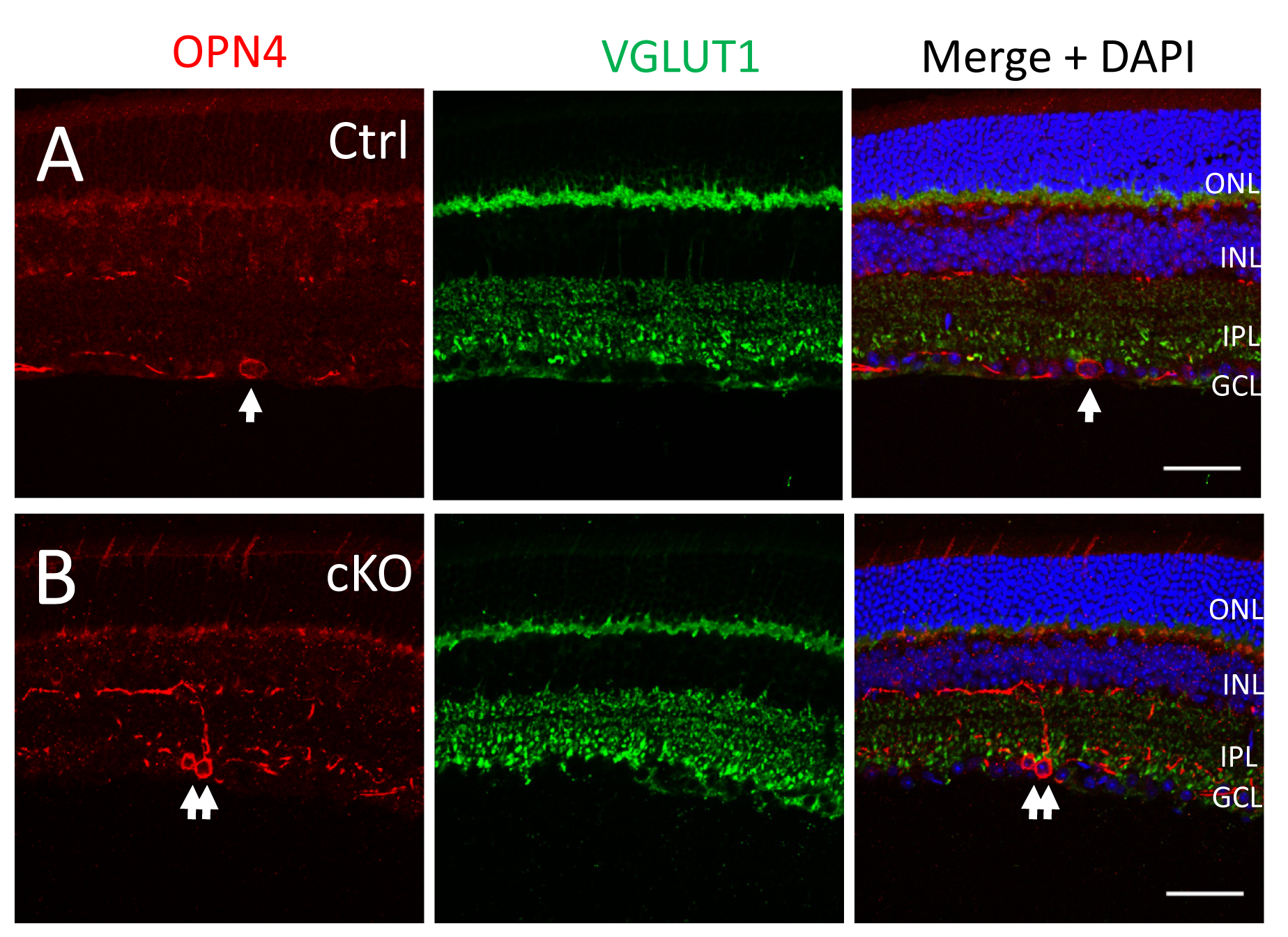

Supplement: Figure S3 — Expression of VGLUT1 is not altered in Vglut2-cKO retinas. Immunostaining for Opn4 and VGLUT1 in (A) control and (B) Vglut2-cKO retinas. Most of VGLUT1 expression is found in the IPL and ONL. VGLUT1 is not found in ipRGCs from either mutant or control retinas (arrows). ONL, outer nuclear layer; INL, inner nuclear layer; IPL, inner plexiform layer; GCL, ganglion cell layer. Scale bar = 50 µm. (TIF) [file pone.0111449.s003.tif]

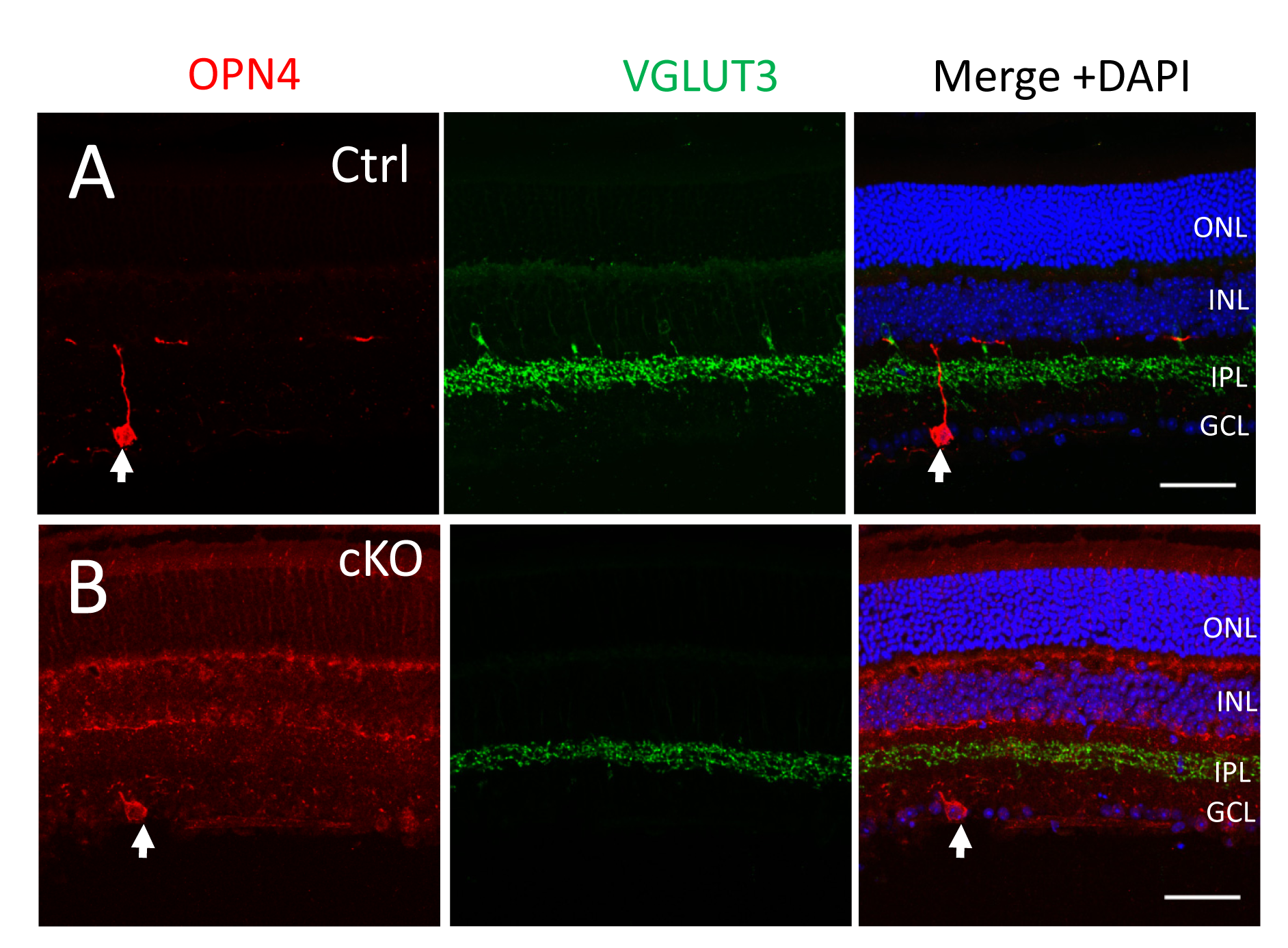

Supplement: Figure S4 — Expression of VGLUT3 is not altered in Vglut2-cKO retinas. Immunostaining for Opn4 and VGLUT3 in (A) control and (B) Vglut2-cKO retinas. Most of VGLUT3 expression is found in the IPL. VGLUT3 is not found in ipRGCs from either mutant or control retinas (arrows). ONL, outer nuclear layer; INL, inner nuclear layer; IPL, inner plexiform layer; GCL, ganglion cell layer. Scale bar = 50 µm. (TIF) [file pone.0111449.s004.tif]
